# Choosing outcomes for a core outcome set: does provision of feedback between delphi survey rounds help prioritise items?

**DOI:** 10.1186/1745-6215-16-S2-P57

**Published:** 2015-11-16

**Authors:** Karen Coulman, Sara Brookes, Katy Chalmers, James Hopkins, Noah Howes, Alex Nicholson, Amanda Owen-Smith, Katie Whale, Jane Blazeby

**Affiliations:** 1 University of Bristol, Bristol, UK; 2 University of Southampton, Southampton, UK; 3 University Hospitals Bristol NHS Trust, Bristol, UK; 4 Taunton and Somerset NHS Trust, Taunton, UK

## Background

A core outcome set (COS) is a minimal set of outcomes to be reported in a trial. Development is often challenging because patients and clinicians identify many important outcomes and prioritisation is difficult. Delphi surveys (with several rounds) are one approach for prioritising outcomes. Summarised participant responses are provided in subsequent ‘rounds’, allowing initial responses to be changed in light of this feedback. While developing a COS for obesity surgery, we explored the impact of this feedback on outcome prioritisation.

## Methods

Systematic reviews and qualitative interviews with patients were undertaken to list all outcomes of obesity surgery. This was operationalised into a 130-item round 1 questionnaire, where participants rated each item on a 1 to 9 scale. Participants were expert clinicians and patients. Round 2 included the same 130 items, the individual's round 1 scores, and the patient and clinician median scores. Participants re-rated each item in light of this feedback. Items rated 8 or 9 by at least 70% of participants were considered ‘important’.

## Results

168 clinicians and 90 patients responded to round 1, and 76% and 90% of these responded to round 2, respectively. In round 1, 18 items were rated ‘important’ by clinicians and 25 by patients. In round 2, these numbers doubled to 36 items for clinicians and 49 for patients.

## Conclusions

In this study, providing feedback did not result in increased prioritisation, but seemed to encourage participants to score more items highly. Further work is needed to understand how participants rate items in light of feedback.

